# Performance of Natural Language Processing Model in Extracting Information from Free-Text Radiology Reports: A Systematic Review and Meta-Analysis

**DOI:** 10.1007/s10278-025-01728-8

**Published:** 2025-10-28

**Authors:** Qingwen Yang, Jiahui Jiang, Xue Dong, Huai Yang, Qiuren Wang, Zhenghan Yang, Dawei Yang, Peng Liu

**Affiliations:** Department of Radiology, Beijing Friendship Hospital, Capital Medical University, No. 95 Yong’an Road, Xicheng District, Beijing, 100050 China

**Keywords:** Natural language processing, Free-text radiology reports, Information extraction, Large language models

## Abstract

The free-text format is widely used in radiology reports for its flexibility of expression; however, its unstructured nature leads to substantial amounts of report data remaining underutilized. A natural language processing (NLP) model for automatic extraction of information from free-text radiology reports can significantly contribute to the development of structured databases, thereby optimizing data utilization. This study aimed to perform a systematic review and meta-analysis that evaluates the performance of NLP systems in extracting information from free-text radiology reports. A systematic literature search was conducted from November 21 to 23, 2024, in PubMed/MEDLINE, Embase, EBSCO, Ovid, Web of Science, and the Cochrane Library. Study quality was assessed using the QUADAS-2 tool. A bivariate random-effects model was applied to obtain the pooled sensitivity, specificity, diagnostic odds ratio (DOR), positive likelihood ratio (PLR), negative likelihood ratio (NLR), and area under the summary receiver operating characteristic curve (AUC). Subgroup analyses (e.g., NLP model types, dataset source, and language types) and a random-effects multivariable meta-regression based on the restricted maximum likelihood (REML) method were conducted to explore potential sources of heterogeneity. Sensitivity analyses (excluding high-risk studies, leave-one-out method, and data integration strategy comparison) were performed to assess the robustness of the findings. A total of 28 studies were included in the final analysis, with 421,692 extracted entities in 51,187 free-text radiology reports. NLP systems achieved high pooled sensitivity (91% [95% CI: 87, 93]) and specificity (96% [95% CI: 93, 97]), with a diagnostic odds ratio of 220 (95% CI: 112, 435) and an area under the curve of 0.98 (95% CI: 0.96, 0.99). Subgroup analysis revealed significantly better performance for extracting single anatomical sites (AUC 0.99; 95% CI: 0.97, 0.99) compared with multiple sites (AUC 0.95; 95% CI: 0.93, 0.97; *p* = 0.001). No significant differences were observed across NLP model types, dataset sources, external validations, languages, or imaging modalities. Multivariable meta-regression further identified anatomical site as the only significant contributor to heterogeneity (coefficient = 2.26; 95% CI: 0.25, 4.27; *p* = 0.027). Sensitivity analyses confirmed the robustness of the findings, and no evidence of publication bias was detected. NLP models demonstrated excellent performance in extracting information from free-text radiology reports. However, the observed heterogeneity highlights the need for enhanced report standardization and improved model generalizability.

**Supplementary Information:**

The online version contains supplementary material available at 10.1007/s10278-025-01728-8.

## Introduction

Radiology reports are essential carriers of information in the field of radiology, serving as a critical bridge for communication between radiologists and clinicians [1]. They play a critical role in clinical decision-making and patient management. Currently, radiology reports are primarily categorized into structured and free-text formats. Due to their flexibility in composition and expressive freedom, free-text radiology reports are widely utilized in clinical practice. However, free-text radiology reports also present challenges, including a lack of standardized terminology, difficulties in information retrieval, and issues such as redundancy or omission of content. These challenges obstruct the efficient extraction and application of information from radiology reports, leaving a substantial portion of retrospective radiology report data underutilized [2].

In recent years, the natural language processing (NLP) model has become an effective tool for the automated extraction of information from free-text radiology reports, facilitating the utilization of such unstructured medical data to some extent [3]. Traditional NLP models extract information from free-text radiology reports using rule-based, machine learning-based, or deep learning-based methods, which require extensive manual annotation for training [4]. With the continuous advancement of deep learning, large language models (LLMs) have emerged. Characterized by their extensive parameterization and pretraining on vast text corpora, LLMs have demonstrated remarkable capabilities in natural language understanding and generation, enabling information extraction from free-text radiology reports without the need for manual annotation [5].

Prior systematic reviews have already highlighted the potential of NLP for radiology reports, but each left important gaps. Pons et al. provided an early overview that summarized a broad set of traditional NLP studies and confirmed their feasibility, yet also emphasized the limited adoption in clinical practice and the lack of external validation [6]. Linna and Kahn updated the field with a larger body of work, showing rising interest in more advanced models and reporting consistently high performance, but they noted that most studies were single-center and descriptive, without quantitative synthesis [7]. Most recently, Kulsoom et al. expanded the scope to include machine learning pipelines and early reports of large language models, and highlighted challenges such as data quality, interpretability, and clinical translation [8]. Nevertheless, these reviews remained primarily descriptive, and none provided a diagnostic test–oriented meta-analysis or systematically compared LLMs with conventional NLP methods. To fill these gaps, the present study conducted a systematic review and meta-analysis, following PRISMA-DTA and informed by CLAIM, to evaluate the diagnostic performance of NLP models—conventional approaches and LLMs—in extracting information from free-text radiology reports.

Advances in NLP models have demonstrated promising performance in extracting information from free-text radiology reports. However, the comparative effectiveness of these models has not yet been systematically evaluated. Therefore, this study aims to systematically review and conduct a meta-analysis to comprehensively evaluate the performance of NLP models in extracting information from free-text radiology reports.

## Materials and Methods

This systematic review and meta-analysis adhered to the Preferred Reporting Items for Systematic Reviews and Meta-Analyses of Diagnostic Test Accuracy Studies statement (i.e., PRISMA-DTA) guidelines and was prospectively registered with the PROSPERO International Prospective Register of Systematic Reviews (No. CRD420251010537) [9]. In addition, the Checklist for Artificial Intelligence in Medical Imaging (CLAIM; 2024 update) was cross-referenced to guide the extraction and reporting of AI-specific items (e.g., data sources, model categories, and external validation) [10].

### Search Strategy

The search strategy was designed based on the PICO criteria[11], which were devised and implemented across multiple databases, including PubMed/MEDLINE, Embase, EBSCO, Ovid, Web of Science, and the Cochrane Library. To ensure a thorough and comprehensive search, as many synonyms of the search terms as possible were considered.

A comprehensive systematic literature search was conducted across six major databases from their inception until November 21 to 23, 2024, during which all searches were completed. The entire search process was documented through screenshots and by exporting search strategies, ensuring methodological transparency and reproducibility. Table 1 summarizes the final search strategies applied in each database. Minor variations were introduced to account for database-specific indexing systems and field restrictions, while maintaining a consistent conceptual scope across databases. Full database-specific search strategies, including stepwise query structures, variations in field codes (e.g., MeSH, Emtree), and Boolean combinations, are provided in Supplementary Table S1.

**Table 1:** Overview of search strategies across databases

Database	Final search strategies
PubMed	(((((((large language model) OR (LLMs)) OR (generative pre-trained transformer)) OR (GPT)) OR (Machine learning)) OR (deep learning)) AND (((structur*) OR (information extraction)) OR (IE))) AND (“radiol* report*")
Embase	‘large language model’/exp OR ‘large language model’ OR ‘lms’ OR ‘generative pre-trained transformer’/exp OR ‘generative pre-trained transformer’ OR ‘gpt’/exp OR ‘gpt’ OR ‘machine learning’/exp OR ‘machine learning’ OR ‘deep learning’/exp OR ‘deep learning’ AND ‘structur*’ OR ‘information extraction’ OR ‘ie’ AND ‘radiol* report*’
Cochrane Library	(“large language model” OR “LLMs” OR “generative pre-trained transformer” OR “GPT” OR “transformer model” OR “foundation model” OR “machine learning” OR “deep learning” OR “artificial intelligence” OR AI):ti,ab,kw (Word variations have been searched) AND (“structured” OR “information extraction” OR “text extraction” OR “structured reporting” OR “IE” OR “data extraction”):ti,ab,kw (Word variations have been searched) AND (radiology NEXT report* OR radiol* NEXT report OR “radiology reporting” OR “radiologic report” OR “medical imaging report” OR “diagnostic imaging” OR imaging NEXT report*):ti,ab,kw (Word variations have been searched)
Web of Science	TS = (“large language model” OR LLMs OR “generative pre-trained transformer” OR GPT OR “machine learning” OR “deep learning”) and Preprint Citation Index (排除–数据库)AND TS = (structur* OR “information extraction” OR IE) and Preprint Citation Index (排除–数据库) AND TS = (“radiol* report*”) and Preprint Citation Index (排除–数据库)
Ovid	(large language model or LLMs or generative pre-trained transformer or GPT or Machine learning or deep learning).mp. [mp = title, book title, abstract, original title, name of substance word, subject heading word, floating sub-heading word, keyword heading word, organism supplementary concept word, protocol supplementary concept word, rare disease supplementary concept word, unique identifier, synonyms, population supplementary concept word, anatomy supplementary concept word] AND (structur* or information extraction or IE).mp. [mp = title, book title, abstract, original title, name of substance word, subject heading word, floating sub-heading word, keyword heading word, organism supplementary concept word, protocol supplementary concept word, rare disease supplementary concept word, unique identifier, synonyms, population supplementary concept word, anatomy supplementary concept word] AND (radiol* report* or imaging report*).mp. [mp = title, book title, abstract, original title, name of substance word, subject heading word, floating sub-heading word, keyword heading word, organism supplementary concept word, protocol supplementary concept word, rare disease supplementary concept word, unique identifier, synonyms, population supplementary concept word, anatomy supplementary concept word]
EBSCO	TI (large language model or LLMs or generative pre-trained transformer or GPT or Machine learning or deep learning) OR AB (large language model or LLMs or generative pre-trained transformer or GPT or Machine learning or deep learning) AND TI (structur* or information extraction or IE) OR AB (structur* or information extraction or IE) AND TI (radiol* report* or imaging report*) OR AB (radiol* report* or imaging report*)

### Study Selection

All retrieved records were imported into EndNote X9 (Thomson Reuters, New York, NY), which was used to identify duplicate articles and to ensure that the screening process was efficient. Additionally, the reference lists of included articles were also manually screened to identify any potentially relevant studies.

The titles and abstracts of the search results were screened by two independent reviewers (with 1 and 5 years of postgraduate experience, respectively), with any inconsistencies resolved through consultation with a third investigator (with 15 years of abdominal imaging experience).

### Eligibility Criteria

Studies were included if they met the following criteria: (1) Study type: Original studies investigating the application of an NLP model in free-text radiology reports. (2) Application domain: Studies specifically focused on extracting information from free-text radiology reports. (3) Model type: Studies that involved either traditional NLP models or large language models (LLMs).

The following study types were excluded: (1) Study format: Articles in the form of reviews, commentaries, case reports, conference abstracts, or those published only as abstracts without an accessible full-text version were excluded. (2) Insufficient data: Studies that lacked essential metrics, making it impossible to calculate a 2 × 2 contingency table.

### Data Extraction and Quality Assessment

Data extraction was conducted by one reviewer and independently verified by a second reviewer. Any discrepancies identified during this process were resolved through discussion until consensus was reached. All 28 included studies were based on free-text radiology reports; none used structured reporting systems.

From each analyzed study, the following data were collected: study details (including author and publication year), number of reports/extracts, NLP model types, dataset, dataset source, external validation, language types, imaging modality-specific reports, extracted anatomical sites, and contingency table data (including true positives, false positives, false negatives, and true negatives). When a study did not directly report a 2 × 2 contingency table, we derived it using the available diagnostic performance metrics provided in the study, such as precision, recall, sample size, or accuracy. In studies that compared multiple NLP models, only the best-performing model was retained for analysis, as identified by the original authors in their main results or conclusions. This approach was taken to reflect the authors’ final recommendation and to avoid introducing subjectivity in model selection. All extracted data were collected in a predefined Excel spreadsheet in Microsoft Excel 2021.

Finally, the risk of bias and methodological quality of the included studies were assessed by two independent reviewers using the Quality Assessment of Diagnostic Accuracy Studies 2 (QUADAS-2) tool [12] in Review Manager 5.3. The QUADAS-2 instrument was specifically developed for evaluating risk of bias in diagnostic accuracy studies. Any discrepancies between the two reviewers were resolved through discussion and, if necessary, adjudicated by a senior radiologist with 15 years of experience in abdominal imaging.

### Model Evolution and Methods Clarification

The included studies reflected the evolution of natural language processing (NLP) methods in radiology during the study period covered by our search (up to November 2024). As summarized in prior reviews [1, 6, 8], early work predominantly relied on rule-based systems and conventional machine learning classifiers, which demonstrated feasibility but were limited in generalizability and external validation. With the increasing availability of larger datasets and computational resources, subsequent studies shifted toward deep learning architectures, achieving higher accuracy but often constrained by single-center data and descriptive evaluations. Most recently, transformer-based and large language models (LLMs) have emerged, offering potential advantages in scalability, generalization, and reduced dependence on manual feature engineering.

For the purpose of synthesis, we categorized the included models into two groups: (1) conventional NLP methods, including rule-based systems, dictionary/lexicon-based pipelines, and classical machine learning algorithms (e.g., naïve Bayes, SVM, logistic regression, CRF, CNN, RNN, BiLSTM, CRNN); (2) transformer-based and large language models (LLMs), encompassing BERT and its derivatives as well as more recent large-scale generative models such as GPT-4 and LLaMA-2. While BERT-type models are sometimes considered pre-LLM architectures, we grouped them with LLMs to reflect the transformer-driven paradigm shift and to enable meaningful comparison with earlier approaches.

### Statistical Analysis

Statistical analysis was performed by an author using commercial software (Stata/SE 18, MIDAS plugin; StataCorp). The MIDAS command was based on a bivariate random-effects model [13, 14]. Inter-study heterogeneity was evaluated using Cochran’s Q test and the *I*^2^ statistic. Due to the presence of multiple 2 × 2 contingency tables resulting from NLP models extracting multiple entities from a report, we applied the micro-average calculation method. The micro-average calculation method aggregates TP, FP, FN, and TN across all entities within each study to construct a composite 2 × 2 contingency table, and then combines these tables from all included studies to calculate the pooled sensitivity, specificity, DOR, and other diagnostic performance metrics. Sensitivity, specificity, diagnostic odds ratio (DOR), the positive likelihood ratio (PLR), negative likelihood ratio (NLR), and the summary receiver operating characteristic curve (SROC) and its area under the curve (AUC) were calculated using the MIDAS command to assess the diagnostic performance of NLP models in extracting information from free-text radiology reports.

Forest plots were generated to visualize the sensitivity and specificity of individual studies, the diagnostic score and odds ratio of individual studies, and the positive likelihood ratio and negative likelihood ratio of individual studies. The Deeks’ funnel plot asymmetry test was performed to evaluate the possible presence of publication bias. To investigate the origin of heterogeneity, subgroup analyses were performed using the following covariates: model types (traditional NLP models [15–29] vs. LLMs [30–42]), dataset source (single center [15–17, 19, 20, 26–32, 34, 36, 40, 42] vs. multi-center [18, 21–25, 33, 35, 37–39, 41]), external validation (yes [22, 24, 25, 32, 35, 37, 39, 41] vs. no [15–21, 23, 26–31, 33, 34, 36, 38, 40, 42]), language type (English only [15–19, 21–28, 30–33, 35, 38, 39, 42] vs. non-English-only [20, 29, 36, 37, 40]), imaging modality-specific reports (single [16–18, 20, 22, 26, 32–35, 37, 40–42] vs. multiple [15, 19, 21, 23–25, 27–30, 36, 38, 39]), and extracted anatomical sites (single [15, 16, 20–22, 25, 28, 32–35, 40–42] vs. multiple [18, 19, 23, 24, 26, 29, 36–39]). A covariate was identified as a potential source of heterogeneity if its subgroup exhibited no significant heterogeneity (*p* > 0.05) and showed a moderate to substantial reduction in *I*^2^ compared to the overall analysis [44]. The *z*-test was used to compare the summary area under the receiver operating characteristic curve (AUC) within the same subgroup.

To further explore the potential sources of heterogeneity that could not be explained by subgroup analysis, a multivariable meta-regression analysis was performed using a random-effects model based on the restricted maximum likelihood (REML) method. Study-level characteristics, including NLP model types, dataset source, external validation, language type, imaging modality specificity, and anatomical site extraction, were included as covariates. Each study contributed a single summary effect size (log DOR), and all variables were entered simultaneously into the model. Regression coefficients, corresponding 95% confidence intervals, residual heterogeneity (*τ*^2^), and pseudo *R*^2^ (reflecting the percentage reduction in *τ*^2^) were reported to evaluate the contribution of each covariate to between-study variability. The reference category for each variable was automatically determined by the software (Stata/SE 18), typically corresponding to the baseline level based on internal coding. Covariates with statistically significant coefficients or those showing a substantial impact on residual heterogeneity were considered potential modifiers of diagnostic accuracy.

### Sensitivity Analysis

Three sensitivity analyses were conducted to assess the robustness of the results. First, studies identified as having a high risk of bias using the QUADAS-2 tool were excluded, and the pooled effect estimates were recalculated to assess the stability of the results. Second, a leave-one-out sensitivity analysis was performed by sequentially removing individual studies to identify potential outliers influencing the overall results. Third, data integration strategy comparison: In the statistical analysis, we applied the micro-average calculation method. Considering that NLP models varied in performance when extracting different entities in each study, we further employed the macro-average calculation method as a supplementary analysis. The macro-average calculation method (1) calculates mean sensitivity, specificity, AUC, PLR, and NLR for each study individually, and (2) weights these study-level metrics based on sample size to obtain the final pooled estimates.

## Result

### Study Selection

**Fig. 1:**
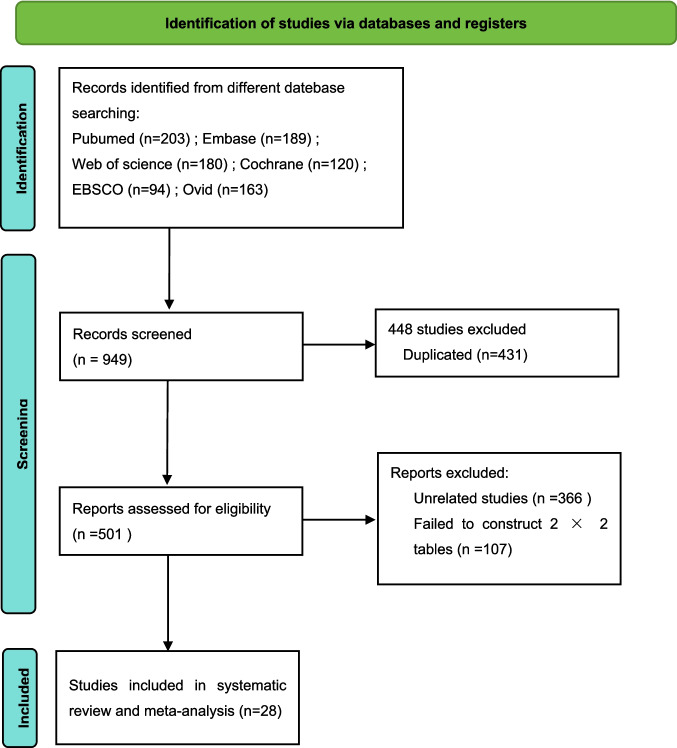
Preferred Reporting Items for Systematic Reviews and Meta-Analyses (PRISMA) flow diagram shows study inclusion and exclusion

The PRISMA flow diagram for this meta-analysis is shown in Fig. 1. A total of 28 studies that strictly met the inclusion and exclusion criteria were ultimately included in this meta-analysis, with 421,692 extracted entities in 51,187 free-text radiology reports.

### Data Extraction and Quality Assessment

Across the 28 included studies, most were conducted in single-center settings, with sample sizes ranging from fewer than 500 to over 100,000 reports. The majority of studies focused on chest and breast imaging, while fewer addressed abdominal or neuroimaging reports. English-language datasets predominated, with only a small proportion of studies using non-English data. Notably, only a minority of studies reported external validation (Table 2). The QUADAS-2 results of the risk of bias and methodological quality assessment of each included study are presented in Fig. S1. Overall, NLP systems for extracting information from free-text radiology reports showed no significant risk of bias in the patient selection, index test, or flow and timing domains. The main concerns were observed in the reference standard domain: two studies were judged to have a high risk of bias due to the lack of a clearly defined reference standard, and 20 studies had an unclear risk of bias because annotator agreement was not reported.

**Table 2:** Characteristics of the included studies

Study	No. of report/extract items	NLP model type	Dataset	Dataset source	External validation	Language types	Imaging modality-specific reports	Extract anatomical sites	TP	FP	FN	TN
Ahumada et al. 2024	67/67	Traditional NLP model	Non-public	Single center	No	Non-English only	Multiple	Multiple	35	1	5	26
Alex et al. 2019	266/12,839	Traditional NLP model	Non-public	Single center	No	English only	Multiple	Single	495	40	23	12281
Arya et al. 2024	7617/38,085	LLMs	Non-public	Single center	No	English only	Multiple	NR	7050	567	567	29901
Bala et al. 2020	409/818	Traditional NLP model	Non-public	Single center	No	English only	Single	Single	76	19	3	720
Brown et al. 2019	628/628	Traditional NLP model	Non-public	Single center	No	English only	Single	NR	136	20	29	443
Chapman et al. 2017	100/747	Traditional NLP model	Public	Multi-center	No	English only	Single	Multiple	220	21	29	447
Cotik et al. 2015	248/248	Traditional NLP model	Non-public	Single center	No	English only	Multiple	Multiple	106	62	21	59
Elmarakeby et al. 2023	14,218/28,436	LLMs	Non-public	Single center	No	English only	NR	NR	3770	1225	1387	22054
Fei et al. 2022	12,022/96,176	Traditional NLP model	Non-public	Single center	No	Non-English only	Single	Single	42140	2710	3706	47620
Fu et al. 2019	333/333	Traditional NLP model	Non-public	Multi-center	No	English only	Multiple	Single	37	0	3	293
Gu et al. 2024	72/648	LLMs	Non-public	Single center	Yes	English only	Single	Single	161	15	19	453
Hassanpour et al. 2016	150/69,400	Traditional NLP model	Non-public	Multi-center	Yes	English only	Single	Single	12073	1807	2295	53225
Hsu et al. 2022	200/2400	LLMs	Non-public	Multi-center	No	English only	Single	Single	318	44	34	2004
Hu et al. 2024	847/6776	LLMs	Non-public	Single center	No	Mix	Single	Single	2072	296	183	4225
Khoruzhaya et al. 2024	2603/2633	LLMs	Non-public	Multi-center	Yes	English only	Single	Single	1284	136	33	1180
Le Guellec et al. 2024	586/586	LLMs	Non-public	Single center	No	English only	Single	Single	514	1	3	68
López-Úbeda et al. 2024	100/100	LLMs	Non-public	Single center	No	Non-English only	Multiple	Multiple	49	20	1	30
Lou et al. 2020	1200/1200	Traditional NLP model	Non-public	Multi-center	No	English only	Multiple	Multiple	70	96	70	964
Meng et al.,2019	480/480	Traditional NLP model	Non-public	Multi-center	Yes	English only	Multiple	Multiple	216	124	24	116
Mithun et al. 2023	501/6003	Traditional NLP model	Both	Multi-center	Yes	English only	Multiple	Single	652	188	221	4942
Park et al. 2022	3362/53,792	Traditional NLP model	Non-public	Single center	No	English only	Single	Multiple	8667	2815	641	41669
Paul et al. 2022	2559/64,224	Traditional NLP model	Non-public	Single center	No	English only	Multiple	NR	11076	1318	1719	50111
Sato et al. 2024	600/2685	LLMs	Non-public	Multi-center	Yes	Non-English only	Single	Multiple	1386	61	30	1208
Su et al.,2024	984/2800	LLMs	Non-public	Multi-center	No	English only	Multiple	Multiple	311	94	85	2310
Tan et al. 2023	98/9702	Traditional NLP model	Non-public	Single center	No	English only	Multiple	Single	826	132	56	8688
Tay et al. 2024	460/8280	LLMs	Non-public	Multi-center	Yes	English only	Multiple	Multiple	601	28	54	7597
Wollek et al. 2024	78/1092	LLMs	Non-public	Single center	No	Non-English only	Single	Single	307	39	41	705
Woźnicki et al. 2024	399/10,514	LLMs	Both	Multi-center	Yes	Mix	Single	Single	1196	483	306	8529

Note: NR, not reported; TP, true positive; FP, false positive; FN, false negative; TN, true negative. “Both” refers to studies that utilized both public and non-public databases

### Statistical Analysis

**Fig. 2:**
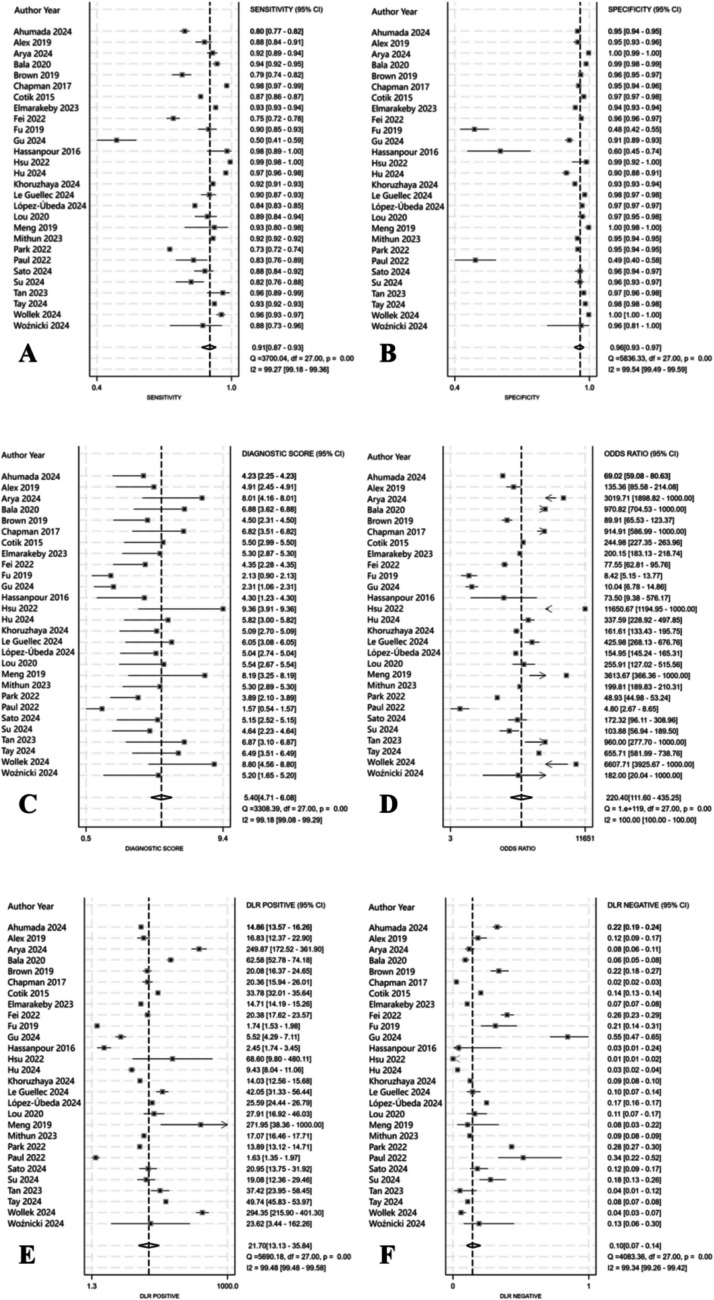
Forest plots of tests. **A** Sensitivity; **B** Specificity; **C** Diagnostic score; **D** Odds ratio; **E** Positive likelihood ratio (PLR); **F** Negative likelihood ratio (NLR)

**Fig. 3:**
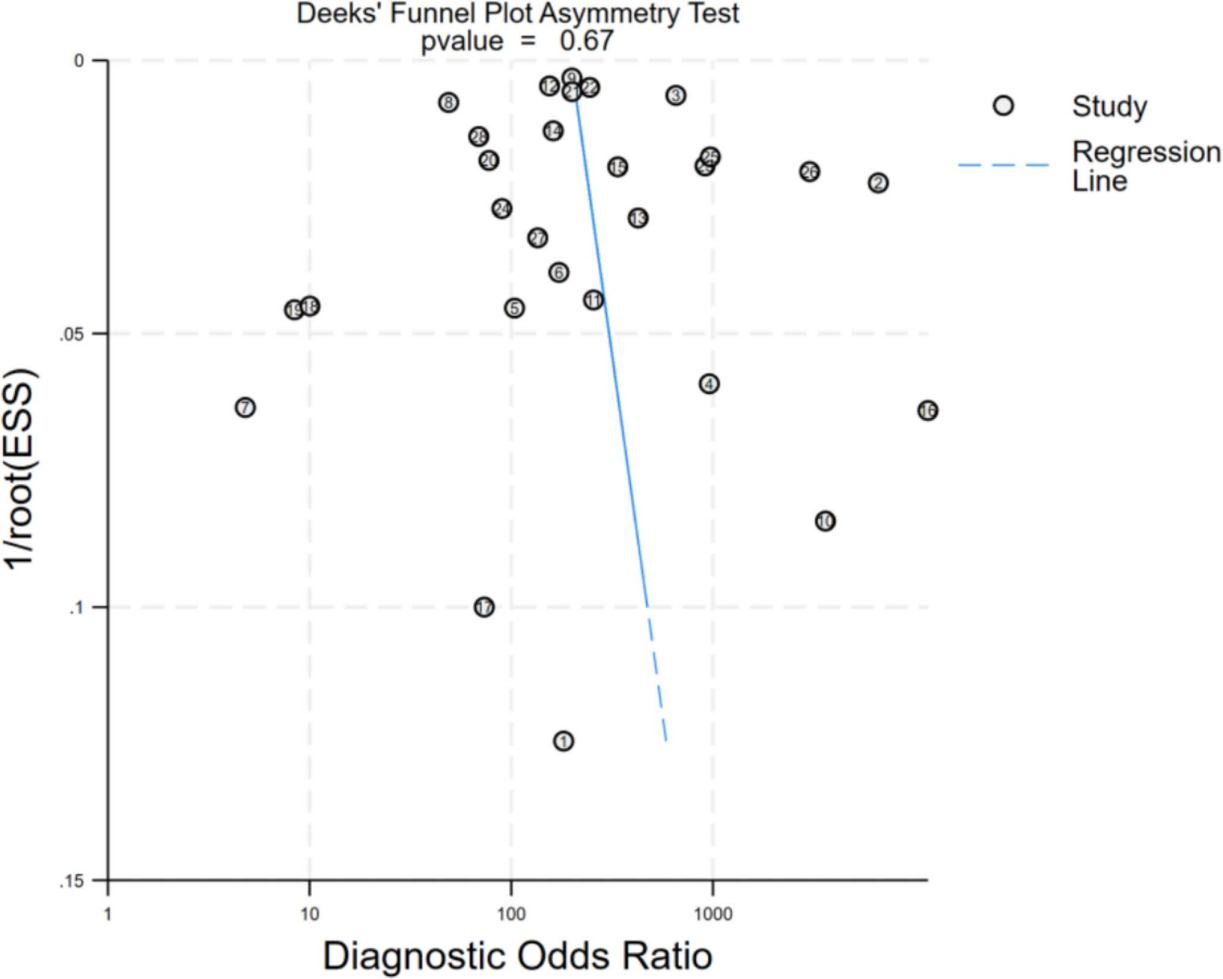
Deeks funnel plot for the publication bias test

The pooled diagnostic performance of NLP systems was excellent, with an overall pooled sensitivity of 91% (95% CI: 87, 93) and pooled specificity of 96% (95% CI: 93, 97). The overall diagnostic odds ratio was high (220; 95% CI: 112–435), and the AUROC reached 0.98 (95% CI: 0.96–0.99), underscoring the robustness of these models (Fig. S2). The positive likelihood ratio (PLR) was 21.7 (95% CI: 13.1, 35.8) and the negative likelihood ratio (NLR) was 0.10 (95% CI: 0.07, 0.14) (Fig. 2). However, substantial heterogeneity was observed across studies (*I*^2^ = 100%; *p* < 0.001). To further investigate, we conducted subgroup analyses and multivariable meta-regression focusing on factors such as NLP model types, dataset source, external validation, language type, imaging modality-specific reports, and extracted anatomical sites. The Deeks’ funnel plot asymmetry test (Fig. 3) indicated no significant publication bias (*p* = 0.67). The symmetrical distribution of study points around the regression line further suggested no small-study effects or systematic bias. Additionally, the regression slope showed no significant deviation, supporting the robustness of the diagnostic odds ratio (DOR) estimates.

### Subgroup Analysis

Subgroup analyses indicated that models extracting information from single anatomical sites achieved superior performance (AUC 0.99; 95% CI: 0.97–0.99) compared with those applied to multiple sites (AUC 0.95; 95% CI: 0.93–0.97; *Z* = 3.51; *p* < 0.001). No significant differences were observed across other subgroup variables, including NLP model type (*p* = 0.06), dataset source (*p* = 0.36), use of external validation (*p* = 0.36), language (*p* > 0.99), or modality-specific reports (*p* = 0.12) (Table 3).

**Table 3:** Subgroup analysis result of natural language processing model in extracting information from free-text radiology report

Subgroup	Studies	*P1*	*I*^2^	Sensitivity (%)	Specificity (%)	Positive likelihood ratio	Negative likelihood ratio	Diagnostic odds ratio	AUROC	*Z*	*P2*
Overall	28	0	100	91 (87, 93)	96 (93, 97)	21.7 (13.1, 35.8)	0.10 (0.07, 0.14)	220 (112, 435)	0.98 (0.96, 0.99)		
NLP model type											
LLMs	13	0	100	90 (85, 93)	97 (94, 98)	26.1 (13.9, 48.9)	0.10 (0.07, 0.15)	251 (104, 604)	0.98 (0.96, 0.99)	1.85	0.06
Traditional NLP model	15	0	100	88 (83, 92)	96 (91, 98)	20.6 (9.5, 44.9)	0.12 (0.08, 0.18)	169 (61, 466)	0.96 (0.94, 0.97)		
Dataset source											
Single-center	17	0	100	92 (88, 94)	96 (92, 98)	21.9 (11.6, 41.1)	0.09 (0.06, 0.13)	249 (105, 590)	0.98 (0.96, 0.99)	0.92	0.36
Multi-center	11	0	100	88 (80, 94)	96 (91, 98)	21.8 (9.4, 50.6)	0.12 (0.07, 0.22)	179 (58, 548)	0.97 (0.95, 0.98)		
External validation											
Yes	8	0	100	91 (84, 95)	95 (87, 98)	18.4 (7.0, 48.1)	0.10 (0.05, 0.17)	190 (60, 603)	0.97 (0.95, 0.98)	0.92	0.36
No	20	0	100	90 (86, 94)	96 (93, 98)	23.4 (13.0, 42.2)	0.10 (0.07, 0.15)	235 (101, 547)	0.98 (0.96, 0.99)		
Language types											
English-only	21	0	100	90 (85, 93)	97 (94, 98)	26.1 (13.9, 48.9)	0.10 (0.07, 0.15)	251 (104, 604)	0.98 (0.96, 0.99)	0	1
Non-English-only	5	0	96	95 (88, 98)	92 (82, 97)	12.4 (5.3, 29.3)	0.06 (0.03, 0.13)	215 (86, 539)	0.98 (0.96, 0.99)		
Imaging modality-specific reports											
Single	14	0	100	93 (89, 95)	95 (94, 96)	20.4 (16.3, 25.4)	0.08 (0.05, 0.12)	269 (161, 450)	0.98 (0.96, 0.99)	1.57	0.12
Multiple	13	0	100	88 (82, 92)	96 (89, 99)	22.1 (7.7, 63.9)	0.12 (0.08, 0.19)	181 (50, 661)	0.96 (0.93, 0.97)		
Extract anatomical sites											
Single	14	0	100	93 (88, 95)	97 (96, 98)	34.2 (20.4, 57.3)	0.08 (0.05, 0.12)	447 (198, 1008)	0.99 (0.97, 0.99)	3.51	0
Multiple	10	0	100	89 (82, 94)	91 (78, 97)	10.3 (3.90, 27.7)	0.11 (0.06, 0.21)	90 (25, 319)	0.95 (0.93, 0.97)		

Note: Data in parentheses are 95% confidence intervals. P1 indicates the*p* value for assessing the general heterogeneity in subgroups by Cochran *Q* test; P2 indicates the *p* value for assessing significant differences in the AUROC between subgroups by the *z*-test

### Multivariable Meta-Regression Analysis

**Fig. 4:**
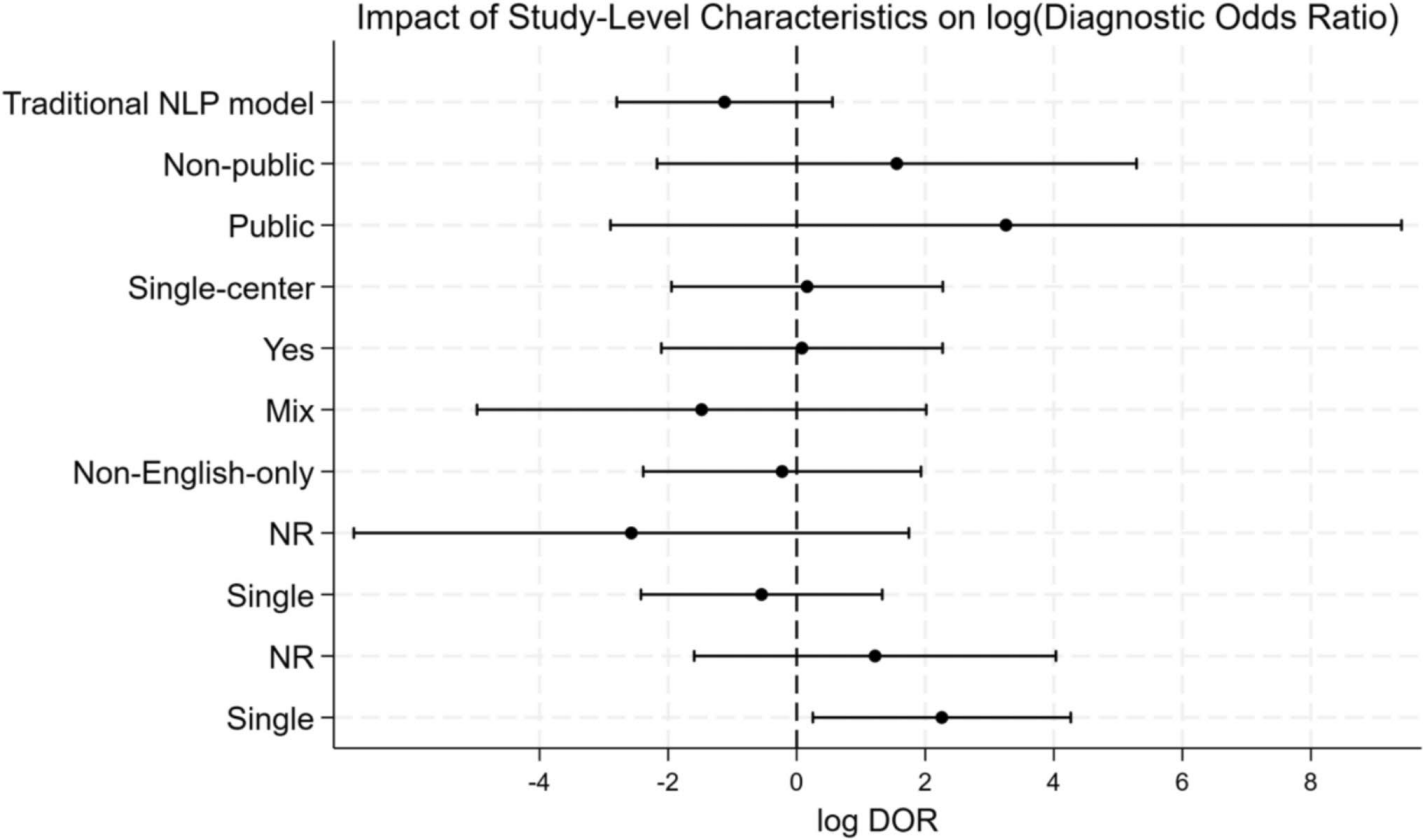
Meta-regression of study-level characteristics on log (diagnostic odds ratio). Each point indicates the estimated effect size (log DOR) for one covariate, with 95% CI. Dotted vertical line indicates the null value (log DOR = 0)

Multivariable meta-regression analysis revealed that none of the included study-level covariates significantly explained the observed heterogeneity across studies (Wald *χ*^2^ = 9.51; *p* = 0.5748; pseudo *R*^2^ = 0%). The residual heterogeneity remained substantial (*τ*^2^ = 3.284; *I*^2^ = 99.01%), indicating that the variability in diagnostic performance could not be fully attributed to the selected covariates. Among all variables, only the extraction of anatomical sites showed a statistically significant association with diagnostic performance: models extracting single anatomical sites exhibited a significantly higher log DOR compared to those extracting multiple sites (coefficient = 2.26; 95% CI: 0.25, 4.27; *p* = 0.027). No statistically significant associations were observed for other covariates, including NLP model types (*p* = 0.190), dataset (*p* = 0.413 and 0.300), dataset source (*p* = 0.880), external validation (*p* = 0.942), language type (*p* = 0.407 and 0.837), and imaging modality-specific reports (*p* = 0.243 and 0.568) (Fig. 4).

### Sensitivity Analysis

After excluding two high-risk studies (due to insufficient reference standard specification), the pooled sensitivity (91%) and specificity (96%) remained unchanged, while the diagnostic odds ratio slightly improved (220 → 247) and confidence intervals narrowed, underscoring result stability (Table 4).

**Table 4:** Sensitivity analysis result after excluding high-risk of bias studies

	No. of studies	Sensitivity (%)	Specificity (%)	Positive likelihood ratio	Negative likelihood ratio	Diagnostic odds ratio	AUROC
Overall	28	91 (87, 93)	96 (93, 97)	21.7 (13.1, 35.8)	0.10 (0.07, 0.14)	220 (112, 435)	0.98 (0.96, 0.99)
Excluding high-risk studies	26	91 (88, 94)	96 (93, 98)	22.2 (13.0, 37.9)	0.09 (0.07, 0.12)	247 (123, 494)	0.98 (0.96, 0.99)

Note: Data in parentheses are 95% confidence intervals. *AUROC*, area under the summary receiver operating characteristic curve

**Fig. 5:**
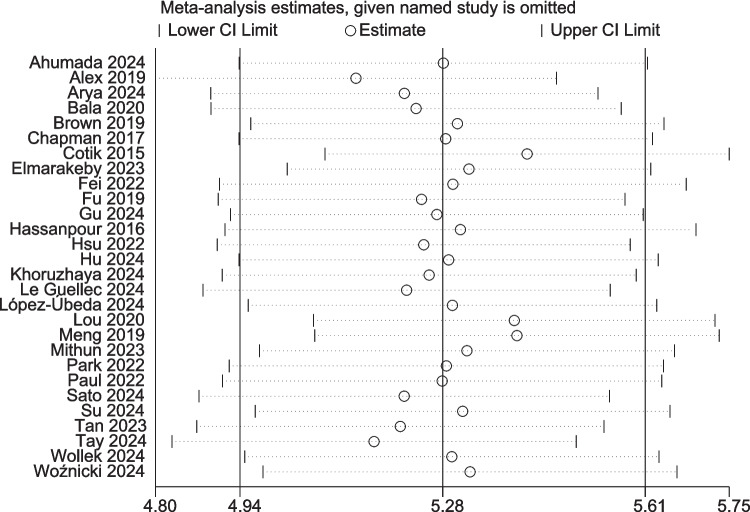
Leave-one-out sensitivity analysis for pooled effect estimate. The circles represent the new pooled estimates after excluding each individual study, while the horizontal bars indicate the 95% confidence intervals (CIs). The vertical solid line represents the original pooled estimate, and the dashed lines denote its corresponding 95% CI

Leave-one-out sensitivity analyses further demonstrated robustness, with pooled DOR estimates ranging from 140 to 272. Notably, excluding Alex 2019 and Woźnicki 2024 produced the largest shifts, reflecting their large sample sizes and influence on overall estimates (Fig. 5).

The micro-average calculation method and macro-average calculation method demonstrated high consistency across diagnostic metrics, reinforcing the robustness of pooled findings even in the presence of heterogeneous datasets (Table S2).

## Discussion

While NLP models have shown significant potential in extracting information from free-text radiology reports, a comprehensive systematic evaluation of their performance remains lacking. To ensure consistency with our study focus, we verified that all included datasets consisted of narrative free-text reports rather than structured formats. Building on this foundation, this meta-analysis synthesizes existing evidence to provide a systematic evaluation of NLP model performance. Our results indicate that NLP models achieve high pooled sensitivity, specificity, and AUC, highlighting their potential clinical applicability in extracting information from free-text radiology reports.

Examining the potential heterogeneity of the meta-analysis was indispensable prior to considering the accuracy of the summary estimates. Substantial heterogeneity (*I*^2^ = 100%) was observed across the included studies, which persisted despite subgroup and meta-regression analyses. Several factors may have contributed to this heterogeneity. Differences in NLP model architecture and training methods may influence information extraction capabilities, while variations in dataset sources may lead to inconsistencies in data quality and annotation standards, affecting model generalizability. Additionally, the absence of external validation could limit the practical applicability of the models, and differences in language type may impact how the models interpret medical terminology. Finally, reports involving multiple anatomical regions tend to exhibit greater text complexity and variability in terminology, increasing the difficulty of accurate information extraction. Importantly, some studies were excluded from subgroup analyses due to discrepancies between their data characteristics and the subgroup classification criteria. While this approach minimized the interference of heterogeneous data and improved the comparability of effect sizes within subgroups, it also reduced the overall sample size, which may have affected statistical power. These limitations highlight the need for methodological standardization, consistent annotation practices, and broader external validation in future research.

In the sensitivity analysis, we applied the macro-average calculation method. This method has several limitations: (1) the dominance effect of large-sample studies may obscure information from smaller studies; (2) it cannot compute a pooled AUC, limiting the overall evaluation of performance and restricting the ability to further investigate potential sources of heterogeneity; (3) it may underestimate inter-study heterogeneity, affecting result interpretability. Therefore, this study did not adopt the macro-average calculation method as the primary statistical approach but instead utilized the micro-average calculation method. By comparing the results of these two methods, we further validated the robustness of the study’s conclusions, ensuring consistency and reliability across different data integration strategies.

This meta-analysis has several limitations. First, high inter-study heterogeneity (I^2^ = 100%) suggests significant methodological or data characteristic differences among included studies, potentially affecting the interpretability of pooled effect estimates. Although subgroup analysis and multivariable meta-regression analysis explored some sources of heterogeneity, it could not fully eliminate this issue. Similar challenges have been reported in prior systematic reviews of NLP in radiology, which highlighted inconsistencies arising from heterogeneous datasets, annotation standards, and lack of reporting standardization [1, 6]. Second, non-externally validated studies were included in this meta-analysis, leading to limitations in generalizability. Prior reviews have similarly emphasized that the lack of external validation and reliance on single-center datasets may constrain the robustness of NLP models [1]. In addition, most included studies evaluated NLP models on English-language radiology reports, which might lead to selection bias. Further research is needed to assess NLP models’ performance across different language contexts. Finally, the selection of a single model per study may have introduced a degree of selection bias, although we minimized subjectivity by following the original authors’ choices.

Future research should focus on enhancing the generalizability and clinical applicability of NLP models. Standardizing and structuring radiology reports, particularly through structured reporting (SR), can reduce variability in terminology and text format, improving model adaptability across institutions, as also recommended in prior reviews [6, 8]. In addition, improving study quality is crucial for future systematic reviews, requiring adherence to TRIPOD guidelines [45], external validation, and minimizing reliance on single-center data to ensure model stability and reliability [1]. Expanding NLP applications to multilingual environments is essential for global applicability, necessitating evaluations in non-English radiology reports [8]. Finally, meta-regression or Bayesian hierarchical models could further explore the impact of data sources, report formats, and model types on heterogeneity, improving the interpretability of pooled effect estimates.

Our findings demonstrate that NLP models achieve high accuracy in extracting information from free-text radiology reports, which has important clinical implications. By improving reliability and consistency in report interpretation, NLP systems could enhance quality control, reduce missed follow-ups, and support timely management of critical findings [20, 23]. Moreover, their ability to process large volumes of narrative data may facilitate integration into routine workflows and enable secondary applications such as clinical decision support and population-level research [1, 6, 8].

## Conclusion

The meta-analysis provides evidence to support the performance of NLP models in extracting information from free-text radiology reports. NLP models demonstrated excellent performance in extracting information from free-text radiology reports. However, the observed heterogeneity highlights the need for enhanced report standardization and improved model generalizability.

## Supplementary Information

Below is the link to the electronic supplementary material.

ESM 1 (DOCX 146 KB)

## Data Availability

Data generated or analyzed during the study are available from the corresponding author upon request.
