# Supplementary material for: Performance of Natural Language Processing Model in Extracting Information from Free-Text Radiology Reports: A Systematic Review and Meta-Analysis
Source: J Imaging Inform Med. 2025 Oct 28;39(4):3639–53. doi: 10.1007/s10278-025-01728-8 (PMC13481926; doi:10.1007/s10278-025-01728-8)
Supplement: Supplementary file 1 [file 10278_2025_1728_MOESM1_ESM.docx]

**Supplemental Materials**

**A**

**B**

**Figure S1: Stacked bar charts of Quality Assessment of Diagnostic Accuracy Studies 2 scores of methodologic study quality that provide an overview of study quality in the whole meta-analysis.**

**Figure S2: Summary receiver operating characteristic (SROC) curve, obtained by using the bivariate model with 95% confidence regions (circles), shows the pooled area under the SROC curve was 0.98 for Natural Language Processing Model in Extracting Information from free-text radiology reports.**

**Table S1: Table S1 presents the complete search strings for PubMed, Embase, Cochrane Library, Web of Science, Ovid, and EBSCO. While the core concepts were consistent, minor adjustments in syntax and indexing were required to accommodate each database’s structure.**

| **Web of Science** | | | |
| --- | --- | --- | --- |
|  | **Search strategy** | **Time** | **Result** |
| #1 | TS=("large language model" OR LLMs OR "generative pre-trained transformer" OR GPT OR "machine learning" OR "deep learning") and Preprint Citation Index (排除–数据库) | Thu Nov 21 2024 11:21:28 | 756713 |
| #2 | TS=(structur* OR "information extraction" OR IE) and Preprint Citation Index (排除–数据库) | Thu Nov 21 2024 11:21:50 | 12147216 |
| #3 | TS=("radiol* report*") and Preprint Citation Index (排除–数据库) | Thu Nov 21 2024 11:22:05 | 5446 |
| #4 | #1 AND #2 AND #3 and Preprint Citation Index (排除–数据库) | Thu Nov 21 2024 11:39:00 | 180 |

| **Cochrance** | | | |
| --- | --- | --- | --- |
|  | **Search strategy** | **Time** | **Result** |
| #1 | ("large language model" OR “LLMs” OR "generative pre-trained transformer" OR “GPT” OR "transformer model" OR "foundation model" OR "machine learning" OR "deep learning" OR "artificial intelligence" OR AI):ti,ab,kw (Word variations have been searched) | 22/11/2024 04:44:42 | 14405 |
| #2 | (“structured” OR "information extraction" OR "text extraction" OR "structured reporting" OR “IE” OR "data extraction"):ti,ab,kw (Word variations have been searched) | 22/11/2024 04:44:42 | 133259 |
| #3 | (radiology NEXT report* OR radiol* NEXT report OR "radiology reporting" OR "radiologic report" OR "medical imaging report" OR "diagnostic imaging" OR imaging NEXT report*):ti,ab,kw (Word variations have been searched) | 22/11/2024 04:44:42 | 45656 |
| #4 | #1 AND #2 AND #3 | 22/11/2024 04:44:42 | 120 |

| **Embase** | | | |
| --- | --- | --- | --- |
|  | **Search strategy** | **Time** | **Result** |
| #1 | 'large language model'/exp OR 'large language model' OR 'llms' OR 'generative pre-trained transformer'/exp OR 'generative pre-trained transformer' OR 'gpt'/exp OR 'gpt' OR 'machine learning'/exp OR 'machine learning' OR 'deep learning'/exp OR 'deep learning' | 2024/11/21 | 752937 |
| #2 | 'structur*' OR 'information extraction' OR 'ie' | 2024/11/21 | 4411631 |
| #3 | 'radiol* report*' | 2024/11/21 | 7976 |
| #4 | #1 AND #2 AND #3 | 2024/11/21 | 189 |

| **Ovid** | | | |
| --- | --- | --- | --- |
|  | **Search strategy** | **Time** | **Result** |
| #1 | (large language model or LLMs or generative pre-trained transformer or GPT or Machine learning or deep learning).mp. [mp=title, book title, abstract, original title, name of substance word, subject heading word, floating sub-heading word, keyword heading word, organism supplementary concept word, protocol supplementary concept word, rare disease supplementary concept word, unique identifier, synonyms, population supplementary concept word, anatomy supplementary concept word] | 1946 to November 22, 2024 | 204749 |
| #2 | (structur* or information extraction or IE).mp. [mp=title, book title, abstract, original title, name of substance word, subject heading word, floating sub-heading word, keyword heading word, organism supplementary concept word, protocol supplementary concept word, rare disease supplementary concept word, unique identifier, synonyms, population supplementary concept word, anatomy supplementary concept word] | 1946 to November 22, 2024 | 3949248 |
| #3 | (radiol* report* or imaging report*).mp. [mp=title, book title, abstract, original title, name of substance word, subject heading word, floating sub-heading word, keyword heading word, organism supplementary concept word, protocol supplementary concept word, rare disease supplementary concept word, unique identifier, synonyms, population supplementary concept word, anatomy supplementary concept word] | 1946 to November 22, 2024 | 11317 |
| #4 | #1 AND #2 AND #3 | 1946 to November 22, 2024 | 163 |

| **EBSCO** | | | |
| --- | --- | --- | --- |
|  | **Search strategy** | **Time** | **Result** |
| S1 | TI ( large language model or LLMs or generative pre-trained transformer or GPT or Machine learning or deep learning ) OR AB ( large language model or LLMs or generative pre-trained transformer or GPT or Machine learning or deep learning ) | 23/11/2024 04:11:12 | 266,914 |
| S2 | TI ( structur* or information extraction or IE ) OR AB ( structur* or information extraction or IE ) | 23/11/2024 04:11:12 | 4,123,155 |
| S3 | TI ( radiol* report* or imaging report* ) OR AB ( radiol* report* or imaging report* ) | 23/11/2024 04:11:12 | 40,280 |
| S4 | S1 AND S2 AND S3 | 23/11/2024 04:11:12 | 167 |

| **Pubmed** | | | |
| --- | --- | --- | --- |
|  | **Search strategy** | **Time** | **Result** |
| #1 | large language model | 21:42:45 | 12259 |
| #2 | LLMs | 21:43:05 | 1556 |
| #3 | generative pre-trained transformer | 21:43:22 | 784 |
| #4 | GPT | 21:43:35 | 6935 |
| #5 | Machine learning | 21:43:51 | 171973 |
| #6 | deep learning | 21:44:05 | 89508 |
| #7 | (((((large language model) OR (LLMs)) OR (generative pre-trained transformer)) OR (GPT)) OR (Machine learning)) OR (deep learning) | 21:52:24 | 232628 |
| #8 | structur* | 21:53:18 | 3390176 |
| #9 | information extraction | 21:53:35 | 265354 |
| #10 | IE | 21:53:56 | 759700 |
| #11 | ((structur*) OR (information extraction)) OR (IE) | 21:54:10 | 4258274 |
| #12 | "radiol* report*" | 21:54:28 | 4594 |
| #13 | (((((((large language model) OR (LLMs)) OR (generative pre-trained transformer)) OR (GPT)) OR (Machine learning)) OR (deep learning)) AND (((structur*) OR (information extraction)) OR (IE))) AND ("radiol* report*") | 21:54:48 | 203 |

| StudyID | Sensitivity | Specificity | DOR | PLR | NLR |
| --- | --- | --- | --- | --- | --- |
| **Macro average calculation method** | | | | | |
| Ahumada 2024 | 0.88 | 0.96 | 182.00 | 23.63 | 0.13 |
| Alex 2019 | 0.84 | 1.00 | 1541.00 | 240.80 | 0.16 |
| Arya 2024 | 0.91 | 0.99 | 717.00 | 61.90 | 0.09 |
| Bala 2020 | 0.96 | 0.97 | 1.00 | 37.98 | 0.04 |
| Brown 2019 | 0.82 | 0.96 | 103.88 | 19.08 | 0.18 |
| Chapman 2017 | 0.89 | 0.94 | 1.10 | 42.19 | 0.11 |
| Cotik 2015 | 0.83 | 0.49 | 4.80 | 1.63 | 0.34 |
| Elmarakeby 2023 | 0.72 | 0.94 | 0.03 | 20.30 | 0.29 |
| Fei 2022 | 0.92 | 0.96 | 271.00 | 22.30 | 0.08 |
| Fu 2019 | 0.93 | 1.00 | 7227.33 | 542.98 | 0.08 |
| Gu 2024 | 0.88 | 0.97 | 234.00 | 28.60 | 0.12 |
| Hassanpour 2016 | 0.83 | 0.97 | 163.00 | 28.40 | 0.17 |
| Hsu 2022 | 0.90 | 0.98 | 459.00 | 48.60 | 0.11 |
| Hu 2024 | 0.94 | 0.96 | 356.00 | 21.40 | 0.06 |
| Khoruzhaya 2024 | 0.97 | 0.90 | 337.59 | 9.43 | 0.03 |
| Le Guellec 2024 | 0.98 | 0.99 | 0.27 | 131.78 | 0.02 |
| López-Úbeda 2024 | 0.98 | 0.60 | 73.50 | 2.45 | 0.03 |
| Lou 2020 | 0.50 | 0.91 | 10.04 | 5.52 | 0.55 |
| Meng 2019 | 0.90 | 0.48 | 8.42 | 1.74 | 0.21 |
| Mithun 2023 | 0.70 | 0.96 | 0.14 | 36.25 | 0.32 |
| Park 2022 | 0.94 | 0.97 | 451.00 | 30.00 | 0.07 |
| Paul 2022 | 0.87 | 0.98 | 267.00 | 34.90 | 0.13 |
| Sato 2024 | 0.97 | 0.96 | 819.00 | 24.40 | 0.03 |
| Su 2024 | 0.79 | 0.96 | 89.91 | 20.08 | 0.22 |
| Tan 2023 | 0.95 | 1.00 | 4592.00 | 251.20 | 0.05 |
| Tay 2024 | 0.90 | 1.00 | 2129.00 | 213.40 | 0.10 |
| Wollek 2024 | 0.88 | 0.96 | 175.00 | 22.30 | 0.13 |
| Woźnicki 2024 | 0.73 | 0.96 | 70.00 | 19.80 | 0.28 |
| Overall | 0.87 | 0.97 | 468.26 | 46.56 | 0.13 |
| **Micro average calculation method** | | | | | |
|  | 0.91 | 0.96 | 220.00 | 21.70 | 0.10 |

**Table S2:** Performance Metrics of Included Studies Using Macro and Micro Average Calculation Methods
